# Outcomes associated with use of makyokansekito, a Japanese herbal kampo medicine, in outpatients with community‐acquired pneumonia: A retrospective cohort study

**DOI:** 10.1002/jgf2.70052

**Published:** 2025-07-27

**Authors:** Yuichiro Matsuo, Takuma Shibahara, Hideo Yasunaga

**Affiliations:** ^1^ Department of Clinical Epidemiology and Health Economics, Graduate School of Medicine The University of Tokyo Bunkyo‐ku Tokyo Japan; ^2^ Department of General Medicine Mie University Tsu Mie Japan

**Keywords:** community‐acquired pneumonia, makyokansekito, outpatients, pharmacoepidemiology

## Abstract

**Introduction:**

Although selected patients with community‐acquired pneumonia (CAP) can be treated in outpatient settings, some exhibit an insufficient response to initial outpatient treatment resulting in subsequent hospitalizations. Laboratory and animal studies have demonstrated that makyokansekito, a Japanese herbal kampo medicine, can alleviate lung damage and inflammation. However, its clinical effectiveness in adult patients with CAP has not been evaluated.

**Methods:**

Using the commercially available JMDC health insurance claims database (Tokyo, Japan), we identified outpatients with CAP between April 2012 and April 2022. Patients were classified into those who received or did not receive makyokansekito on the day of diagnosis. The primary outcome was hospitalization within 30 days. The secondary outcomes included antibiotic treatment duration and total medical costs. Multivariate regression analyses were used to compare the outcomes between the two groups.

**Results:**

Among 76,177 eligible patients, 273 and 75,904 were classified into the makyokansekito and non‐makyokansekito groups, respectively. After adjustment, the proportions of hospitalized patients in the makyokansekito and non‐makyokansekito groups were 3.0 and 3.4%, respectively, with a difference of −0.4% (95% confidence interval [CI], −2.5% to 1.8%; *p* = 0.705). The adjusted mean antibiotic treatment durations were 6.3 and 6.5 days, respectively, with a difference of −0.2 days (95% CI, −0.6% to 0.1%; *p* = 0.155). Adjusted total medical costs were 53,455 and 52,000 Japanese yen (JPY), respectively, with a difference of 1452 JPY (95% CI, −10,988 to 18,525 JPY; *p* = 0.852).

**Conclusion:**

The use of makyokansekito in outpatients with CAP was not associated with a reduction in hospitalization.

## 1 INTRODUCTION

Outpatient management with oral antibiotics is a feasible option for selected patients with community‐acquired pneumonia (CAP) who have relatively mild symptoms and few comorbidities.^1^ Outpatient treatment is associated with lower cost and higher patient satisfaction compared with inpatient management.^2^, ^3^ However, some patients exhibit an insufficient response to initial outpatient treatment, leading to prolonged antibiotic treatment and subsequent hospitalization.^3^, ^4^, ^5^ The short‐term hospitalization rate of outpatients with CAP has been reported to range from approximately 2%–10%.^4^, ^5^, ^6^ The primary reasons for hospitalization include worsening of pneumonia‐related symptoms and exacerbation of existing comorbidities,^3^, ^7^ which may potentially be prevented by treatment that modifies the clinical course of pneumonia. An insufficient clinical response has also been associated with increased healthcare costs.^8^

Antibiotic therapy targeting identified or suspected pathogens remains the cornerstone of pneumonia treatment. Several adjunctive therapies have been investigated, including glucocorticoids, vitamin supplementation, statins, and anticoagulants, but none have demonstrated a risk‐benefit balance justifying their routine use in outpatients with CAP.^9^, ^10^, ^11^

Kampo medicine, or traditional Japanese herbal medicine, is widely used in Japan to treat a variety of diseases, often as a complement to Western medicine.^12^, ^13^, ^14^, ^15^, ^16^ Kampo medicine has also been used for the treatment of CAP,^17^, ^18^, ^19^ primarily to alleviate the associated symptoms. Makyokansekito (“maxing shigan” in Chinese), a kampo formulation, contains four herbal ingredients: ephedra, gypsum, armeniacae, and glycyrrhizae. According to kampo theories, the combination of ephedra and gypsum is expected to reduce “heat” in the lungs, while the combination of ephedra and armeniacae is thought to promote pulmonary clearance. Glycyrrhizae is added to harmonize the overall formulation.^20^ The use of makyokansekito is considered most suitable for “young” and “robust” patients, consistent with the kampo medicine concept of *jitsu‐sho* (*sho* is a concept similar to “status”).^21^ Therefore, it may be a promising adjunctive therapy that could potentially modulate the clinical course of CAP in younger outpatients. Laboratory and animal studies have demonstrated that makyokansekito can alleviate lung damage and inflammation.^22^, ^23^, ^24^ It has also been used for the treatment of respiratory diseases including infections in several Asian countries.^25^, ^26^, ^27^ However, its clinical effectiveness in adult patients with CAP has not been evaluated.

We aimed to examine whether the use of makyokansekito in outpatients with CAP is associated with a reduction in subsequent hospitalizations, using a large claims database in Japan.

## 2 METHODS

### 2.1 Study design and data source

We conducted a retrospective cohort study using the commercially available JMDC health insurance claims database (Tokyo, Japan).^28^ This database contains claims data from January 2005 to May 2022 and complete prescription data from April 2012, covering approximately 10 million insured individuals in Japan, who are primarily employees of Japanese companies and their families. The database includes administrative claims data from both outpatient visits and hospital admissions, with diagnoses recorded according to the International Classification of Diseases, 10th revision (ICD‐10) codes and medications recorded according to the World Health Organization Anatomical Therapeutic Chemical classification system.

This study was approved by the Institutional Review Board of the authors' institution. Given the anonymized nature of the data, the requirement for written informed consent was waived.

### 2.2 Patients

We initially identified patients aged 18–65 years who had been diagnosed with bacterial pneumonia between April 2012 and April 2022. The diagnosis was confirmed based on a combination of diagnostic codes (ICD‐10 codes: J13, J14, J15, J16, J18, or A481), performance of either a radiograph or computed tomography on the day of diagnosis (index date), and initiation of antibiotic treatment within 2 days of the index date. Patients with a history of pneumonia episodes within the preceding 6 months were excluded. The study population was further restricted to those diagnosed in the outpatient setting, who were not hospitalized on the index date, and who were prescribed one of the guideline‐recommended outpatient treatments for CAP in Japan on the index date, specifically oral beta‐lactams, oral fluoroquinolones, or oral macrolides.^29^

Patients with specific conditions or complications on the index date were excluded, including aspiration pneumonia, lung abscess, empyema, pneumothorax, pulmonary tuberculosis, fungal pulmonary infection, exacerbation of chronic obstructive pulmonary disease, asthma attack, and pleural effusion. We also excluded those with a history of interstitial pneumonia, pregnancy, neutropenia, or human immunodeficiency virus infection, defined by the presence of a recorded diagnosis within 6 months prior to the index date. Additional exclusions were applied to patients with a history of hospitalization within the previous 90 days, those who had received intravenous antibiotics within the previous 90 days, and those who had received oral antibiotics within the previous 7 days. We also excluded patients diagnosed with influenza or coronavirus disease 2019 (COVID‐19) within the previous 30 days, including the index date. Additionally, we excluded patients who had received makyokansekito within the previous 90 days. Finally, to ensure a sufficient look‐back period for baseline assessment, we only included patients who had been recorded in the database for at least 6 months prior to the index date and whose index date was in July 2012 or later, allowing for at least 3 months of complete prescription data before the index date. Individuals were included in the study cohort multiple times if they met the eligibility criteria more than once. The details of the disease, procedure, and drug definitions are provided in Table S1.

### 2.3 Exposure variable

The exposure variable was defined as the receipt of makyokansekito on the index date. Makyokansekito is available as packaged granules in Japan.^30^, ^31^

### 2.4 Outcomes

The primary outcome was hospitalization within 30 days following the index date. The secondary outcomes included antibiotic treatment duration and total medical costs. Antibiotic treatment duration was defined as the number of consecutive days covered by antibiotic prescriptions from the index date. To avoid extreme values, treatment durations exceeding 14 days were capped at 14 days. This upper limit was selected because some patients with CAP may receive treatment for up to 14 days depending on the clinical presentation.^32^ Durations exceeding 14 days were assumed to reflect potential complications (e.g., empyema and lung abscesses) requiring prolonged treatment, and differences beyond this threshold were considered clinically less meaningful. Total medical costs were calculated as direct medical costs, including both outpatient and inpatient expenses incurred on the index date and within the subsequent 30 days. These costs were expressed in Japanese yen (JPY; 1 US Dollar = approximately 110 JPY as of 2021).^33^

Patients were followed up until the completion of the 30‐day follow‐up period, death, or loss to follow‐up due to a change in health insurer, whichever occurred first. The outcomes of patients lost to follow up before the completion of the 30‐day period were based on the information available during their follow‐up period.

### 2.5 Adjustment variables

The following patient characteristics were collected and included as adjustment variables in the analyses: age (categorized into 18–35, 36–45, 46–55, and 56–65 years), gender, year of the index date (categorized into 2012–2015, 2016–2019, and 2020–2022), body mass index (BMI) (categorized into <18.5, 18.5–24.9, 25.0–29.9, and ≥ 30 kg/m^2^), smoking status (current smoker or not), and baseline comorbidities, defined as the presence of a recorded diagnosis within 6 months prior to the index date, assessed by the Charlson Comorbidity Index (CCI)^34^ (categorized into 0, 1, and ≥2). Healthcare utilization metrics included the number of months with administrative claims records in the previous 6 months (categorized into 0, 1, 2, and ≥3), which serve as a proxy for healthcare facility visits, and healthcare costs incurred in the previous 6 months (categorized into 0, 1–4999, 5000–49,999, and ≥50,000 JPY). We also collected data on the diagnosis of atypical pneumonia and the prescription of the following antibiotics on the index date: intravenous antibiotics, oral beta‐lactams, oral fluoroquinolones, and oral macrolides. Additionally, prescriptions for oral nonsteroidal anti‐inflammatory drugs, oral acetaminophen, and cough suppressants or expectorants were collected as proxies of baseline symptom severity. The details of the disease and drug definitions are provided in Table S1.

### 2.6 Statistical analysis

First, we described the characteristics of the patients who received or did not receive makyokansekito on the index date.

Second, we examined the association between makyokansekito use and the outcomes using multivariate outcome regression analyses. We estimated the proportion of hospitalization in the makyokansekito and non‐makyokansekito groups using a logistic regression model. Antibiotic treatment duration was estimated using generalized linear models with a log link function and negative binomial distribution. The total medical costs were estimated using generalized linear models with a log link function and gamma distribution. All regression models included the above‐mentioned adjustment variables, and generalized estimation equations were applied to account for clustering within the treating medical facilities.^35^ Missing values for BMI (34.0% missing) and smoking status (35.2%) were treated as separate categories. The estimated values were standardized over the entire population to obtain the marginal mean outcomes in the makyokansekito and non‐makyokansekito groups.^36^ The means and the differences between groups were calculated, and their 95% confidence intervals (CIs) were calculated with nonparametric bootstrapping using 1000 iterations.^37^ Secondary outcome analyses were considered exploratory, and no adjustments for multiple comparisons were applied.

### 2.7 Sensitivity analyses

We performed two sensitivity analyses. First, given that only a limited number of patients received makyokansekito (273, 0.36% of the total sample), our results may have been influenced by non‐causal associations related to physician practice patterns. To address this issue, we restricted the study population to patients treated in medical institutions with a history of prescribing makyokansekito within the study population. Second, we excluded those who were prescribed any other form of kampo medicine on the index date. These sensitivity analyses were conducted using the same statistical methods as the main analyses, except that the number of bootstrapping iterations used to calculate the CIs was reduced to 200 because of limitations in computational resources.

## 3 RESULTS

### 3.1 Patients

**FIGURE 1:**
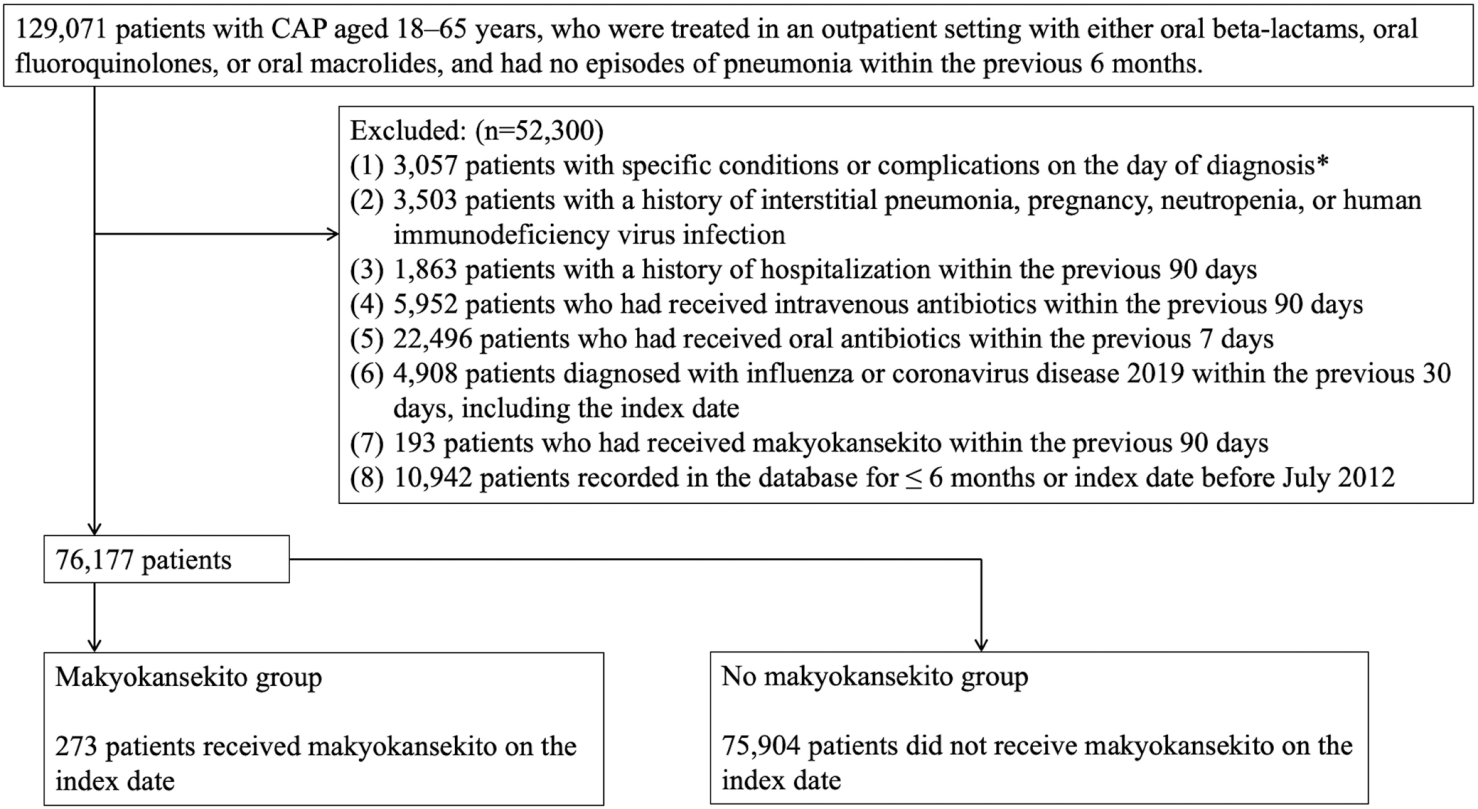
Flowchart of selection of the study participants. *Includes aspiration pneumonia, lung abscess, empyema, pneumothorax, pulmonary tuberculosis, fungal pulmonary infection, exacerbation of chronic obstructive pulmonary disease, asthma attack, and pleural effusion.

We initially identified 129,071 patients with CAP between April 2014 and April 2022 aged 18 to 65 years, who were treated in an outpatient setting with either oral beta‐lactams, oral fluoroquinolones, or oral macrolides, and had not experienced any episodes of pneumonia within the previous 6 months. After applying the exclusion criteria, 76,177 patients (from 72,675 distinct individuals) remained for the final analysis. Among them, 273 and 75,904 patients were classified into the makyokansekito and non‐makyokansekito groups, respectively (Figure 1). Six (0.01%) patients died within their 30‐day follow‐up period, and 480 (0.63%) were lost to follow‐up because of a change in health insurer. All other patients completed their 30‐day follow‐up.

### 3.2 Baseline characteristics

The mean age of the study patients was 43 years, and 55% were male. A total of 66% of the patients had no comorbidities as assessed by the CCI. The baseline characteristics of the patients in the makyokansekito and non‐makyokansekito groups were generally similar, except that patients in the makyokansekito group were more likely to have atypical pneumonia, less likely to receive oral beta‐lactams, and more likely to receive oral macrolides (Table 1).

**TABLE 1:** Baseline characteristics of the patients.

	Overall, *n* = 76,177	Makyokansekito group, *n* = 273	Non‐makyokansekito group, *n* = 75,904
Age, years			
Mean (SD)	42.8 (12.1)	41.2 (11.2)	42.8 (12.1)
18–35	22,267 (29.2%)	90 (33.0%)	22,177 (29.2%)
36–45	22,321 (29.3%)	93 (34.1%)	22,228 (29.3%)
46–55	17,166 (22.5%)	53 (19.4%)	17,113 (22.5%)
56–65	14,423 (18.9%)	37 (13.6%)	14,386 (19.0%)
Gender, male	42,123 (55.3%)	153 (56.0%)	41,970 (55.3%)
Year			
2012–2015	14,358 (18.8%)	42 (15.4%)	14,316 (18.9%)
2016–2019	45,710 (60.0%)	168 (61.5%)	45,542 (60.0%)
2020–2022	16,109 (21.1%)	63 (23.1%)	16,046 (21.1%)
Body mass index (kg/m^2^)			
<18.5	5637 (7.4%)	13 (4.8%)	5624 (7.4%)
18.5–24.9	31,953 (41.9%)	110 (40.3%)	31,843 (42.0%)
25.0–29.9	9525 (12.5%)	39 (14.3%)	9486 (12.5%)
≥30	2765 (3.6%)	14 (5.1%)	2751 (3.6%)
Missing	26,297 (34.5%)	97 (35.5%)	26,200 (34.5%)
Smoking status			
No	35,743 (46.9%)	138 (50.5%)	35,605 (46.9%)
Yes	12,743 (16.7%)	30 (11.0%)	12,713 (16.7%)
Missing	27,691 (36.4%)	105 (38.5%)	27,586 (36.3%)
Charlson comorbidity index			
0	50,043 (65.7%)	186 (68.1%)	49,857 (65.7%)
1	18,743 (24.6%)	64 (23.4%)	18,679 (24.6%)
≥2	7391 (9.7%)	23 (8.4%)	7368 (9.7%)
Number of months of administrative claim records in the previous 6 months			
0	15,749 (20.7%)	56 (20.5%)	15,693 (20.7%)
1	14,889 (19.5%)	61 (22.3%)	14,828 (19.5%)
2	11,785 (15.5%)	50 (18.3%)	11,735 (15.5%)
≥3	33,754 (44.3%)	106 (38.8%)	33,648 (44.3%)
Healthcare costs in the previous 6 months (JPY)			
0	19,893 (26.1%)	72 (26.4%)	19,821 (26.1%)
1–4999	5193 (6.8%)	30 (11.0%)	5163 (6.8%)
5000–49,999	32,482 (42.6%)	110 (40.3%)	32,372 (42.6%)
≥50,000	18,609 (24.4%)	61 (22.3%)	18,548 (24.4%)
Atypical pneumonia	7080 (9.3%)	75 (27.5%)	7005 (9.2%)
Intravenous antibiotics	12,794 (16.8%)	31 (11.4%)	12,763 (16.8%)
Oral beta‐lactams	12,499 (16.4%)	14 (5.1%)	12,485 (16.4%)
Oral fluoroquinolones	37,793 (49.6%)	132 (48.4%)	37,661 (49.6%)
Oral macrolides	28,503 (37.4%)	133 (48.7%)	28,370 (37.4%)
Oral nonsteroidal anti‐inflammatory drugs	13,429 (17.6%)	44 (16.1%)	13,385 (17.6%)
Oral acetaminophen	25,759 (33.8%)	71 (26.0%)	25,688 (33.8%)
Cough suppressants or expectorants	55,851 (73.3%)	201 (73.6%)	55,650 (73.3%)

*Note*: Dichotomous and categorical variables are reported as numbers and percentages.

Abbreviations: JPY, Japanese yen; SD, standard deviation.

### 3.3 Outcomes

A total of 6 patients (2.2%) in the makyokansekito group and 2551 patients (3.4%) in the non‐makyokansekito group were hospitalized within 30 days. The adjusted proportion of patients who were hospitalized was 3.0% in the makyokansekito group and 3.4% in the non‐makyokansekito group, with a difference of −0.4% (95% CI, −2.5% to 1.8%; *p* = 0.705).

A total of nine patients (3.3%) in the makyokansekito group and 2813 patients (3.7%) in the non‐makyokansekito group were treated with antibiotics for more than 14 days; their antibiotic treatment durations were capped at 14 days for the analysis. The adjusted mean antibiotic treatment duration was 6.3 days in the makyokansekito group and 6.5 days in the non‐makyokansekito group, with a difference of −0.2 days (95% CI, −0.6 to 0.1%; *p* = 0.155). The adjusted medical cost was 53,455 JPY in the makyokansekito group and 52,000 JPY in the non‐makyokansekito group, with a difference of 1452 JPY (95% CI, −10,988 to 18,525 JPY; *p* = 0.155) (Table 2).

**TABLE 2:** Main results.

	Crude		Adjusted		
	Makyokansekito group	Non‐makyokansekito group	Makyokansekito group (95% CI)	Non‐makyokansekito group (95% CI)	Difference (95% CI); *p*
*Primary outcome*					
Hospitalization	6 (2.2%)	2551 (3.4%)	3.0% (0.9%–5.2%)	3.4% (3.3%–3.5%)	−0.4% (−2.5 to 1.8%); *p* = 0.705
*Secondary outcomes*					
Antibiotic treatment duration (days)	5.8	6.5	6.3 (6.0–6.6)	6.5 (6.5–6.5)	−0.2 (−0.6 to 0.1); *p* = 0.155
Total medical costs	43,989 JPY	52,250 JPY	53,455 JPY (41,021–71,121 JPY)	52,000 JPY (51,269–53,152 JPY)	1452 JPY (−10,988 to 18,525 JPY); *p* = 0.852

*Note*: The means of the outcomes for each group are presented.

Abbreviations: CI, confidence interval; JPY, Japanese yen.

### 3.4 Sensitivity analyses

Restricting the study population to patients treated in medical institutions with a history of prescribing makyokansekito yielded 1637 patients (273 in the makyokansekito group and 1364 in the non‐makyokansekito group) from 168 institutions (originally 17,417 institutions). The patient characteristics in each group were similar to those of the original population; namely, patients in the makyokansekito group were more likely to have atypical pneumonia and less likely to receive oral beta‐lactams (Table S2). Outcome analyses within this subpopulation yielded results that were qualitatively consistent with those of the main analyses (Table 3). A total of 50 patients (18.3%) in the makyokansekito group and 5851 patients (7.7%) in the non‐makyokansekito group received some other kampo medicine on the index date. After excluding these patients, the results remained qualitatively unchanged (Table 4).

**TABLE 3:** Results of the sensitivity analyses restricting the study population to patients treated in medical institutions with a history of prescribing makyokansekito.

	Crude		Adjusted		
	Makyokansekito group	Non‐makyokansekito group	Makyokansekito group (95% CI)	Non‐makyokansekito group (95% CI)	Difference (95% CI); *p*
*Primary outcome*					
Hospitalization	6 (2.2%)	29 (2.1%)	2.6% (0.8–5.3%)	2.1% (1.3–3.0%)	0.5% (−1.6 to 3.3%); *p* = 0.733
*Secondary outcomes*					
Antibiotic treatment duration (days)	5.8	5.8	6.0 (5.7–6.4)	6.2 (6.0–6.4)	−0.1 (−0.5 to 0.2); *p* = 0.440
Total medical costs	43,989 JPY	43,871 JPY	47,259 JPY (38,128–60,352 JPY)	42,337 JPY (39,376–46,189 JPY)	4923 JPY (−3870 to 17,636 JPY); *p* = 0.329

*Note*: The means of the outcomes for each group are presented.

Abbreviations: CI, confidence interval; JPY, Japanese yen.

**TABLE 4:** Results of the sensitivity analyses excluding patients prescribed any kampo medicine other than makyokansekito on the index date.

	Crude		Adjusted		
	Makyokansekito group	Non‐makyokansekito group	Makyokansekito group (95% CI)	Non‐makyokansekito group (95% CI)	Difference (95% CI); *p*
*Primary outcome*					
Hospitalization	6 (2.7%)	2434 (3.5%)	3.8% (1.3%–7.9%)	3.5% (3.3%–3.6%)	0.3% (−2.2 to 4.5%); *p* = 0.875
*Secondary outcomes*					
Antibiotic treatment duration (days)	5.8	6.6	6.4 (6.0 to 6.8)	6.6 (6.5 to 6.6)	−0.2 (−0.5 to 0.3); *p* = 0.410
Total medical costs	45,128 JPY	52,782 JPY	55,002 JPY (41,142–73,331 JPY)	52,546 JPY (51,641–53,665 JPY)	2455 JPY (−11,090 to 21,311 JPY); *p* = 0.775

*Note*: The means of the outcomes for each group are presented.

Abbreviations: CI, confidence interval; JPY, Japanese yen.

## 4 DISCUSSION

In this observational study using a large Japanese claims database, the use of makyokansekito was not associated with a reduction in hospitalization, antibiotic treatment duration, or total medical cost among outpatients with CAP.

To the best of our knowledge, this is the first study to assess the clinical effect of makyokansekito in patients with CAP. One small observational study examined the use of makyokansekito in patients with COVID‐19 pneumonia and reported that makyokansekito use was associated with a shorter time to symptom relief.^26^ Similarly, in our study, patients in the makyokansekito group may have experienced more rapid symptom relief, which we were unable to assess. However, any such benefit may not have translated into improvement in the outcomes (hospitalization, antibiotic duration, or total medical costs) in our population, which consisted of young, low‐risk patients with few comorbidities.

Our study does not support the routine use of makyokansekito to prevent hospitalization in outpatients with CAP. Moreover, although serious adverse events associated with makyokansekito use are rare, they have been reported.^38^ Therefore, its use should at least be limited to patients most likely to benefit from it. In particular, it is important to consider each patient's *sho* and to use makyokansekito primarily in individuals whose clinical presentation is consistent with *jitsu‐sho*, for which makyokansekito is considered appropriate.^20^

Our study has several limitations. First, the diagnosis of CAP using diagnostic codes in outpatient claims data has not been validated in Japan. To enhance the specificity of the diagnosis, we used a combination of imaging studies and antibiotic use.^39^ Second, culture test results were not available, hindering identification of the reasons for the insufficient response to initial outpatient treatment. Third, detailed clinical data such as vital signs, laboratory data, and symptoms were not available in the database. Nevertheless, we restricted our study population to outpatients with CAP and excluded those with pulmonary complications and adjusted for the use of symptomatic medications as proxies for baseline symptom severity. Therefore, bias because of disease severity was likely minimal.

In conclusion, the use of makyokansekito in outpatients with CAP was not associated with a reduction in hospitalization, antibiotic treatment duration, or total medical cost.

## AUTHOR CONTRIBUTIONS

**Yuichiro Matsuo:** Conceptualization; formal analysis; visualization; writing – original draft; methodology; writing – review and editing. **Takuma Shibahara:** Conceptualization; writing – review and editing. **Hideo Yasunaga:** Writing – review and editing; supervision; project administration.

## FUNDING INFORMATION

This work was supported by grants from the Ministry of Health, Labour, and Welfare of Japan (grant number 23AA2003), and grants from the Japan Kampo Medicines Manufacturers Association (Grant on Health Economics Research, 2024).

## CONFLICT OF INTEREST STATEMENT

The authors declare no conflicts of interest in conducting this research and preparing the article.

## ETHICS STATEMENT

This study was approved by the Institutional Review Board of the University of Tokyo (approval number, 10862‐(1); approval date June 13, 2018).

## PATIENT CONSENT STATEMENT

Given the anonymized nature of the data, the requirement for written informed consent was waived.

## Supporting information


Table S1.
Table S2.

## Data Availability

The datasets analyzed during the current study are not publicly available because of contracts with the data provider (JMDC).
