# Supplementary material for: Outcomes associated with use of makyokansekito, a Japanese herbal kampo medicine, in outpatients with community‐acquired pneumonia: A retrospective cohort study
Source: J Gen Fam Med. 2025 Jul 27;26(5):427–34. doi: 10.1002/jgf2.70052 (PMC12404173; doi:10.1002/jgf2.70052)
Supplement: Supplementary file 1 — Table S1. Table S2. [file JGF2-26-427-s001.docx]

**Supplemental Table 1** Definitions of diseases, procedures, and drugs

|  | Definitions |
| --- | --- |
| Diseases |  |
| Bacterial pneumonia  (including atypical pneumonia) | J13–J16, J18, A481 |
| Atypical pneumonia | J157, J160, A481 |
| Aspiration pneumonia | J69 |
| Lung abscess | J65 |
| Empyema | J86 |
| Pneumothorax | J93 |
| Pulmonary tuberculosis | A15, A16 |
| Exacerbation of COPD | J441 |
| Asthma attack | J46 |
| Pleural effusion | J90 |
| Interstitial pneumonia | J70, J84, M051 M321, M330, M331, M351; each combined with the diagnosis name including "pneumonia" |
| Pregnancy | O00–O99 |
| Neutropenia | D070 |
| Human immunodeficiency virus infection | B20–B24, R75 |
| Influenza | J09–J11 |
| COVID-19 | U071, U072 |
|  |  |
| Diseases included in the Charlson Comorbidity Index |  |
| Myocardial infarction | I21–I23, I252 |
| Congestive cardiac failure | I11, I13, I255, I42, I43, I50, I517 |
| Peripheral vascular disease | I70–I73, I770, I771, K551, K558, K559, R02, Z958, Z959 |
| Cerebrovascular disease | G45, G46, I60–T69 |
| Dementia | A810, F00–F03, F051, G30, G31 |
| Chronic pulmonary disease | I26, I27, J40–J47, J60–J67, J684, J701, J703 |
| Rheumatological disease | M05, M06 M09, M120, M315, M32–M36 |
| Liver disease | B18, I85, I864, I982, K70, K71, K721, K729, K76, R162, Z944 |
| Diabetes mellitus | E10–E14 |
| Hemiplegia or paraplegia | G114, G81–G83 |
| Renal disease | I12, I13, N01, N03, N05, N07, N08, N171, N172, N18, N19, Z49, Z940, Z992 |
| Malignancy | C00–C43, C45–C76, C80–C85, C88–97 |
| Metastatic solid tumor | C77–C79 |
| Human immunodeficiency virus infection | B20–B24 |
|  |  |
| Procedures |  |
| Radiographs | E001, E002 |
| Computed tomography | E200, E203 |
|  |  |
| Drugs |  |
| All antibiotics | J01 |
| Beta-lactams | J01C, J01D |
| Fluoroquinolones | J01M |
| Macrolides | J01FA |
| Nonsteroidal anti-inflammatory drugs | M01A |
| Acetaminophen | N02BE01 |
| Cough suppressants or expectorants | R05C, R05D, R05F |

Variables were defined based on the presence of the codes listed above. Diseases were identified using International Classification of Diseases, 10th revision (ICD- 10) codes, procedures were identified using Japanese original K-codes, and drugs were identified using World Health Organization Anatomical Therapeutic Chemical (WHO-ATC) codes. Makyokansekito does not have a specific WHO-ATC code and was extracted based on the drug name.

COPD, chronic obstructive pulmonary disease; COVID-19, coronavirus disease 2019.

**Supplemental Table 2** Baseline characteristics of the patients, restricted to those treated at medical institutions with a history of prescribing makyokansekito

|  | Overall n=1,637 | Makyokansekito group n=273 | Non-makyokansekito group n=1,364 |
| --- | --- | --- | --- |
| Age, years |  |  |  |
| mean (SD) | 42.1 (11.6) | 41.2 (11.2) | 42.3 (11.6) |
| 18–35 | 497 (30.4%) | 90 (33.0%) | 407 (29.8%) |
| 36–45 | 528 (32.3%) | 93 (34.1%) | 435 (31.9%) |
| 46–55 | 363 (22.2%) | 53 (19.4%) | 310 (22.7%) |
| 56–65 | 249 (15.2%) | 37 (13.6%) | 212 (15.5%) |
| Sex, male | 854 (52.2%) | 153 (56.0%) | 701 (51.4%) |
| Year |  |  |  |
| 2012–2015 | 205 (12.5%) | 42 (15.4%) | 163 (12.0%) |
| 2016–2019 | 1,016 (62.1%) | 168 (61.5%) | 848 (62.2%) |
| 2020–2022 | 416 (25.4%) | 63 (23.1%) | 353 (25.9%) |
| Body mass index (kg/m^2^) |  |  |  |
| < 18.5 | 114 (7.0%) | 13 (4.8%) | 101 (7.4%) |
| 18.5–24.9 | 718 (43.9%) | 110 (40.3%) | 608 (44.6%) |
| 25.0–29.9 | 193 (11.8%) | 39 (14.3%) | 154 (11.3%) |
| ≥ 30 | 55 (3.4%) | 14 (5.1%) | 41 (3.0%) |
| Missing | 557 (34.0%) | 97 (35.5%) | 460 (33.7%) |
| Smoking status |  |  |  |
| No | 828 (50.6%) | 138 (50.5%) | 690 (50.6%) |
| Yes | 232 (14.2%) | 30 (11.0%) | 202 (14.8%) |
| Missing | 577 (35.2%) | 105 (38.5%) | 472 (34.6%) |
| Charlson comorbidity index |  |  |  |
| 0 | 991 (60.5%) | 186 (68.1%) | 805 (59.0%) |
| 1 | 500 (30.5%) | 64 (23.4%) | 436 (32.0%) |
| ≥ 2 | 146 (8.9%) | 23 (8.4%) | 123 (9.0%) |
| Number of months of administrative claim records in the previous 6 months |  |  |  |
| 0 | 317 (19.4%) | 56 (20.5%) | 261 (19.1%) |
| 1 | 329 (20.1%) | 61 (22.3%) | 268 (19.6%) |
| 2 | 264 (16.1%) | 50 (18.3%) | 214 (15.7%) |
| ≥ 3 | 727 (44.4%) | 106 (38.8%) | 621 (45.5%) |
| Healthcare costs in the previous 6 months (JPY) |  |  |  |
| 0 | 417 (25.5%) | 72 (26.4%) | 345 (25.3%) |
| 1–4,999 | 111 (6.8%) | 30 (11.0%) | 81 (5.9%) |
| 5,000–49,999 | 698 (42.6%) | 110 (40.3%) | 588 (43.1%) |
| ≥ 50,000 | 411 (25.1%) | 61 (22.3%) | 350 (25.7%) |
| Atypical pneumonia | 266 (16.2%) | 75 (27.5%) | 191 (14.0%) |
| Intravenous antibiotics | 186 (11.4%) | 31 (11.4%) | 155 (11.4%) |
| Oral beta-lactams | 193 (11.8%) | 14 (5.1%) | 179 (13.1%) |
| Oral fluoroquinolones | 758 (46.3%) | 132 (48.4%) | 626 (45.9%) |
| Oral macrolides | 735 (44.9%) | 133 (48.7%) | 602 (44.1%) |
| Oral nonsteroidal anti-inflammatory drugs | 209 (12.8%) | 44 (16.1%) | 165 (12.1%) |
| Oral acetaminophen | 401 (24.5%) | 71 (26.0%) | 330 (24.2%) |
| Cough suppressants or expectorants | 1,130 (69.0%) | 201 (73.6%) | 929 (68.1%) |

Dichotomous and categorical variables are reported as numbers and percentages.

SD, standard deviation; JPY, Japanese yen.
